# From Choledocholithiasis to Metastatic Pancreatic Adenocarcinoma: A Diagnostic Odyssey Highlighting Missed Red Flags

**DOI:** 10.7759/cureus.116897

**Published:** 2026-09-25

**Authors:** Abdullah Farooq, Jazeela Saud, Henok E Sisay, Husnain Hanif, Muhammad Subhan, Alwaleed Alfadhli

**Affiliations:** 1 Medicine, Allama Iqbal Medical College, Lahore, PAK; 2 Internal Medicine, East Medical Ward, Mayo Hospital, Lahore, PAK; 3 Anesthesiology, Thumbay Group, Ajman, ARE; 4 Medicine, Nawaz Sharif Medical College, Gujrat, PAK; 5 Internal Medicine, Jinnah Hospital Lahore, Allama Iqbal Medical College, Lahore, PAK; 6 College of Medicine, Royal College of Surgeons in Ireland, Dublin, IRL

**Keywords:** ca 19-9, case report, common bile duct stricture, ercp, obstructive jaundice, pancreatic adenocarcinoma

## Abstract

Pancreatic adenocarcinoma is one of the deadliest types of cancer, with a very gloomy prognosis, especially if the cancer is at an advanced stage at the time of diagnosis. Among young adults, the vague symptoms that are usual with pancreatic cancer can be confused with those of a benign biliary condition, so the diagnosis is often late. It may be quite challenging to recognize that a bile duct obstruction with jaundice is due to a periampullary tumor, which is similar to a bile duct stone, unless one suspects it very strongly. We present the case of a 38-year-old man who suffered from increasing abdominal pain, significant weight loss, and obstructive jaundice. An initial triphasic CT showed the presence of dilated intrahepatic biliary ducts and a dilated common bile duct (CBD) with duodenal spots that were thought to be passed gallstones. At the same time, a liver lesion was identified as a probable hemangioma. The diagnosis was postponed even though the liver function tests were progressively deteriorating (total bilirubin increased to 10.4 mg/dL and alkaline phosphatase to 1394 U/L). Further endoscopic retrograde cholangiopancreatography (ERCP) revealed a distal CBD stricture without stones; therefore, a plastic stent was placed. No tissue sampling was obtained during ERCP. Five months later, an extremely high carbohydrate antigen 19-9 (CA 19-9) (>500 U/mL) and a repeat triphasic CT showed a 43 × 45 mm pancreatic head tumor with liver and lung metastases. These findings supported a clinical-radiological diagnosis of metastatic pancreatic malignancy, presumed pancreatic adenocarcinoma; histopathological confirmation of the pancreatic or hepatic lesions was not obtained. Subsequent ascitic fluid cytology showed malignant cells consistent with adenocarcinoma, but immunocytochemistry was not available, so the pancreatic primary was not independently established. This case highlights that if a biliary stricture occurs without a stone that can be identified in a patient with progressive obstructive jaundice, the stricture should be considered malignant until demonstrated otherwise. Early obtaining of tissue for diagnosis and appropriate use of tumor markers as supportive rather than definitive tests are the main elements to steer clear of a long diagnostic odyssey.

## Introduction

Pancreatic ductal adenocarcinoma (PDAC) remains one of the deadliest gastrointestinal malignancies, with a five-year survival rate of approximately 12% across all stages [1]. For patients presenting with metastatic disease (stage IV), the prognosis is particularly dismal, with a median survival of 6-11 months and a five-year survival rate below 3% [2]. While PDAC typically presents in the sixth to seventh decade of life, approximately 1-2% of cases occur in individuals under 45 years of age [3]. In younger patients, the diagnosis is frequently delayed due to a lower initial clinical suspicion for malignancy, often leading to more advanced disease at presentation and worse outcomes [4].

Obstructive jaundice is a hallmark of periampullary tumors but is also a common presentation of choledocholithiasis, creating a diagnostic dilemma that can result in significant delays in cancer diagnosis [5]. Differentiating a benign stone from a malignant stricture is critical, as a delay of even a few weeks can affect resectability and survival [5]. Serum carbohydrate antigen 19-9 (CA 19-9) is the most widely utilized tumor marker for PDAC [6]. In patients with obstructive jaundice, a CA 19-9 level exceeding 500 U/mL has been reported in some series to have a positive predictive value greater than 95% for pancreatic malignancy; however, this estimate must be interpreted cautiously because benign cholangitis and cholestasis can also markedly elevate CA 19-9. CA 19-9 should therefore be regarded as a supportive adjunct to imaging and tissue diagnosis, not as a stand-alone diagnostic test [6,7]. Endoscopic retrograde cholangiopancreatography (ERCP) serves a dual role in both diagnosis and palliation of biliary obstruction [8]. However, when a malignant-appearing biliary stricture is encountered in the absence of an identifiable stone, tissue sampling via brush cytology, intraductal biopsy, or endoscopic ultrasound-guided fine-needle aspiration is essential, despite their modest sensitivities (30-70%) [8,9]. The failure to obtain tissue at the time of stricture identification is a critical diagnostic error with significant clinical consequences [10]. An indeterminate distal biliary stricture should be distinguished from a clearly malignant stricture, and ERCP-based sampling and endoscopic ultrasound (EUS)-guided tissue acquisition should be considered complementary rather than mutually exclusive.

This case report describes a 38-year-old male whose initial presentation of obstructive jaundice was attributed to passed gallstones, leading to a five-month diagnostic odyssey. The case is unique for several reasons: (1) the patient’s young age, which contributed to a lower suspicion for malignancy; (2) discordant imaging findings between ultrasound and CT, including a liver lesion initially mischaracterized as a hemangioma and the interval development of ascites; (3) the performance of ERCP with stent placement without tissue acquisition, despite a clear malignant-appearing distal common bile duct (CBD) stricture; and (4) a markedly elevated CA 19-9 (>500 U/mL) that was obtained only after clinical deterioration.

The objectives of this report are (1) to highlight the diagnostic challenges in distinguishing malignant biliary strictures from choledocholithiasis, particularly in younger populations; (2) to emphasize the supportive, non-definitive role of early tumor marker evaluation (CA 19-9) in patients with unexplained biliary obstruction; (3) to underscore the necessity of tissue acquisition during ERCP when a stricture is encountered without a stone; and (4) to provide a practical diagnostic algorithm to guide clinicians in similar presentations. Through this case, we aim to educate clinicians on recognizing red flags that should prompt earlier consideration of pancreatic malignancy and to advocate for a more aggressive diagnostic approach in patients with persistent or worsening obstructive jaundice.

## Case presentation

A 38-year-old male, married father of four children and a textile merchant by occupation, presented to the hepatology outpatient department on Day 1 with a three-month history of progressive abdominal pain, unintentional weight loss of approximately 8 kg, intermittent undocumented fever, generalized pruritus, and a burning sensation during micturition. One month prior to presentation, he developed bilateral pitting pedal edema and reported progressive yellowish discoloration of his skin and sclera. His past medical history was unremarkable, with no prior diagnosis of diabetes mellitus, hypertension, or chronic liver or kidney disease. However, clinical notes indicated that blood sugar monitoring (BSR) charting was initiated during his admission, raising suspicion for possible new-onset diabetes mellitus, a well-recognized paraneoplastic phenomenon associated with pancreatic malignancies [11,12]. No objective glucose or HbA1c values were available in the records; therefore, new-onset diabetes was suspected but not established. He was a non-smoker, did not consume alcohol, had no known drug allergies, and reported no family history of gastrointestinal or pancreatic malignancies.

On initial physical examination, the patient was alert, conscious, and oriented with a Glasgow Coma Scale of 15/15. His vital signs were stable: blood pressure 130/80 mmHg, pulse 88 beats per minute, respiratory rate 14 breaths per minute, and he was afebrile with an SpO_2_ of 98% on room air. General examination revealed bilateral pitting pedal edema; clinically evident scleral icterus was not clearly documented at this visit but appeared after one week. No pallor, cyanosis, clubbing, or palpable lymphadenopathy was noted. Cardiovascular examination revealed normal S1 and S2 sounds with no murmurs, and respiratory examination demonstrated normal vesicular breathing bilaterally with no added sounds. Abdominal examination showed generalized tenderness, more pronounced in the epigastrium and right upper quadrant, with voluntary guarding that limited deep palpation; no distinct masses or organomegaly was appreciated, and bowel sounds were normoactive. Initial laboratory investigations performed on the same day revealed a cholestatic pattern with markedly elevated alkaline phosphatase (810 U/L; normal ≤130), gamma-glutamyl transferase (664 U/L; normal ≤60), and mild transaminitis (alanine aminotransferase (ALT) 141 U/L, aspartate aminotransferase (AST) 66 U/L), while total bilirubin remained within normal limits (0.5 mg/dL) and renal function was normal (creatinine 0.8 mg/dL) (Table 1).

**Table 1:** Laboratory Investigations ALT: alanine aminotransferase; SGPT: serum glutamic-pyruvic transaminase; AST: aspartate aminotransferase; SGOT: serum glutamic-oxaloacetic transaminase; Gamma-GT: gamma-glutamyl transferase; A:G: albumin-to-globulin ratio; CA 19-9: carbohydrate antigen 19-9

Test	Day 1	Day 25	Reference Range	Unit
Liver Function Tests				
Total Bilirubin	0.5	10.4	≤1.2	mg/dL
Direct Bilirubin	0.3	9.6	0.1-0.3	mg/dL
Indirect Bilirubin	0.2	0.8	0.1-1.0	mg/dL
ALT (SGPT)	141	70	≤41	U/L
AST (SGOT)	66	79	≤40	U/L
Gamma-GT	664	463	≤60	U/L
Alkaline Phosphatase	810	1394	40-130	U/L
Total Protein	7.1	6.7	6.6-8.7	g/dL
Albumin	4.1	2.9	3.5-5.0	g/dL
Globulins	3.0	3.8	2.3-3.5	g/dL
A:G Ratio	1.4	0.8	0.8-2.0	-
Renal Function Tests				
Creatinine	0.8	N/A	0.7-1.4	mg/dL
Urea	13	N/A	8-43	mg/dL
Blood Urea Nitrogen	5.9	N/A	4-20	mg/dL
Uric Acid	2.7	N/A	3.4-7.0	mg/dL
Tumor Markers				
CA 19-9	N/A	>500.0	<37	U/mL
Alpha-Fetoprotein	N/A	1.98	0.0-6.05	IU/mL

Given the clinical picture of obstructive jaundice, a triphasic CT of the abdomen and pelvis was performed on Day 14. This demonstrated dilatation of the intrahepatic biliary channels and a dilated CBD measuring 16 mm at the porta hepatis, extending down to the ampulla of Vater. Multiple dense intraluminal foci measuring 11 to 16 mm were noted in the duodenum, which were interpreted as likely passed gallstones. A well-defined, rounded focal lesion measuring 19 × 18 mm was observed in segment II of the left lobe of the liver, exhibiting hypodensity on the arterial phase with incomplete delayed-phase fill-in, leading to a radiological diagnosis of a probable hemangioma. The pancreas appeared unremarkable with no pancreatic duct dilatation, and no abdominopelvic ascites was detected (Figure 1).

**Figure 1:**
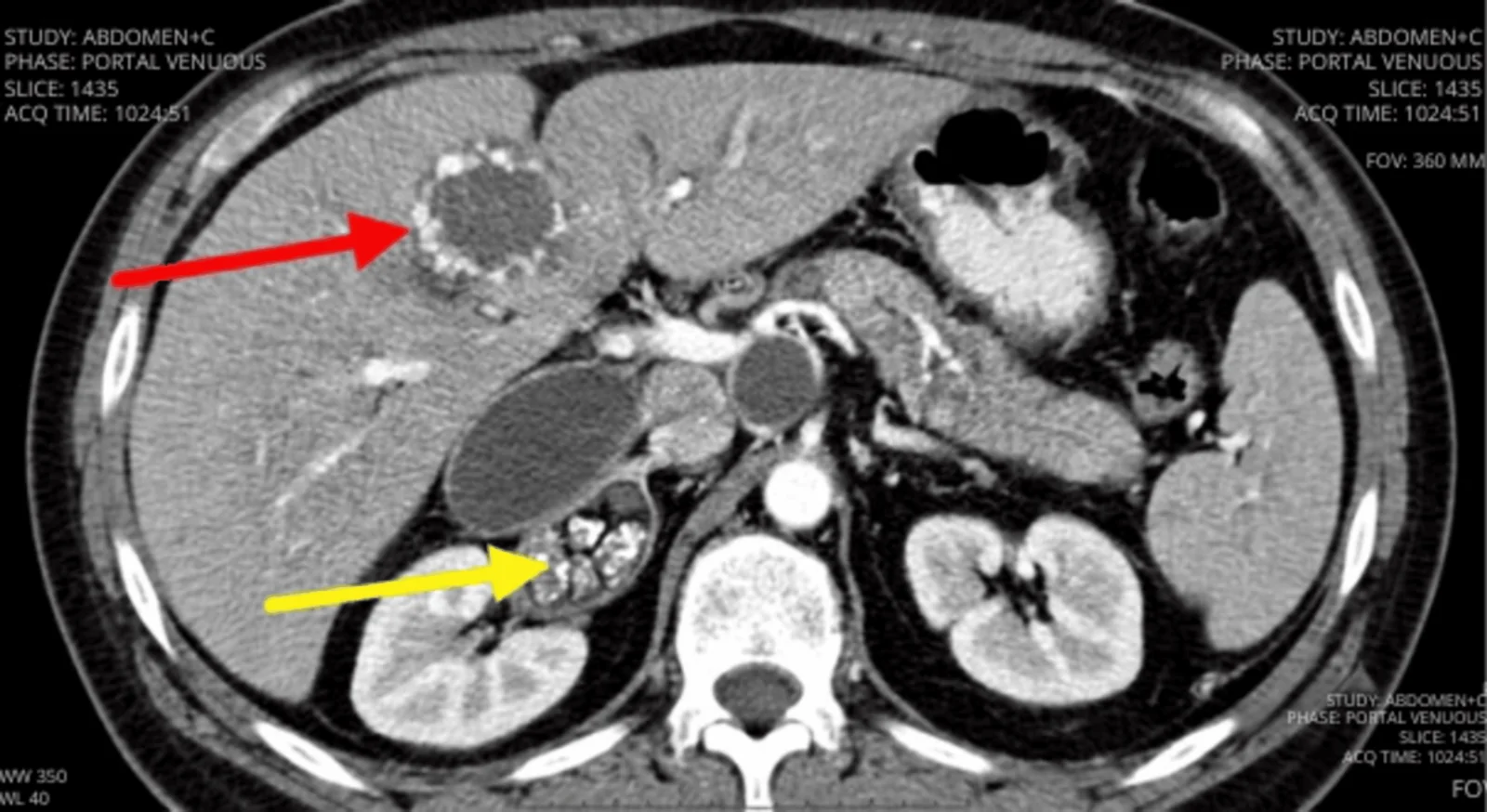
Initial Triphasic CT Abdomen Axial contrast-enhanced CT image (portal venous phase) demonstrating marked dilatation of the common bile duct (16 mm) extending to the ampulla. Multiple hyperdense intraluminal foci are noted in the duodenum (yellow arrow), which were initially interpreted as passed gallstones. A well-circumscribed 19 × 18 mm hypodense lesion in segment II of the left hepatic lobe (red arrow) exhibits peripheral enhancement, initially characterized as a probable hemangioma. No ascites is seen at this stage.

However, the patient's clinical condition continued to deteriorate, and repeat laboratory tests on Day 25 revealed dramatic worsening: total bilirubin had risen to 10.4 mg/dL, direct bilirubin to 9.6 mg/dL, alkaline phosphatase to 1394 U/L, and albumin had dropped to 2.9 g/dL from 4.1 g/dL just 25 days earlier (Table 1). Concurrent ultrasound of the abdomen performed on the same day noted a well-defined heterogeneous focal lesion with irregular margins in segment III of the left lobe of the liver, measuring 3.4 × 2.3 cm-distinctly different in location (segment III versus segment II) and characteristics from the CT lesion, raising the possibility of either a second separate lesion or mischaracterization of a malignant deposit. The ultrasound also revealed a clump of gallstones (up to 9 mm) with echogenic sludge and wall echogenic foci suggestive of adenomyomatosis; bilateral increased renal echogenicity with poor corticomedullary differentiation consistent with Grade II nephropathy; and, importantly, mild-to-moderate volume abdominopelvic ascites - a new finding that had developed within just 11 days of the prior CT that had shown no ascites (Figure 2).

**Figure 2:**
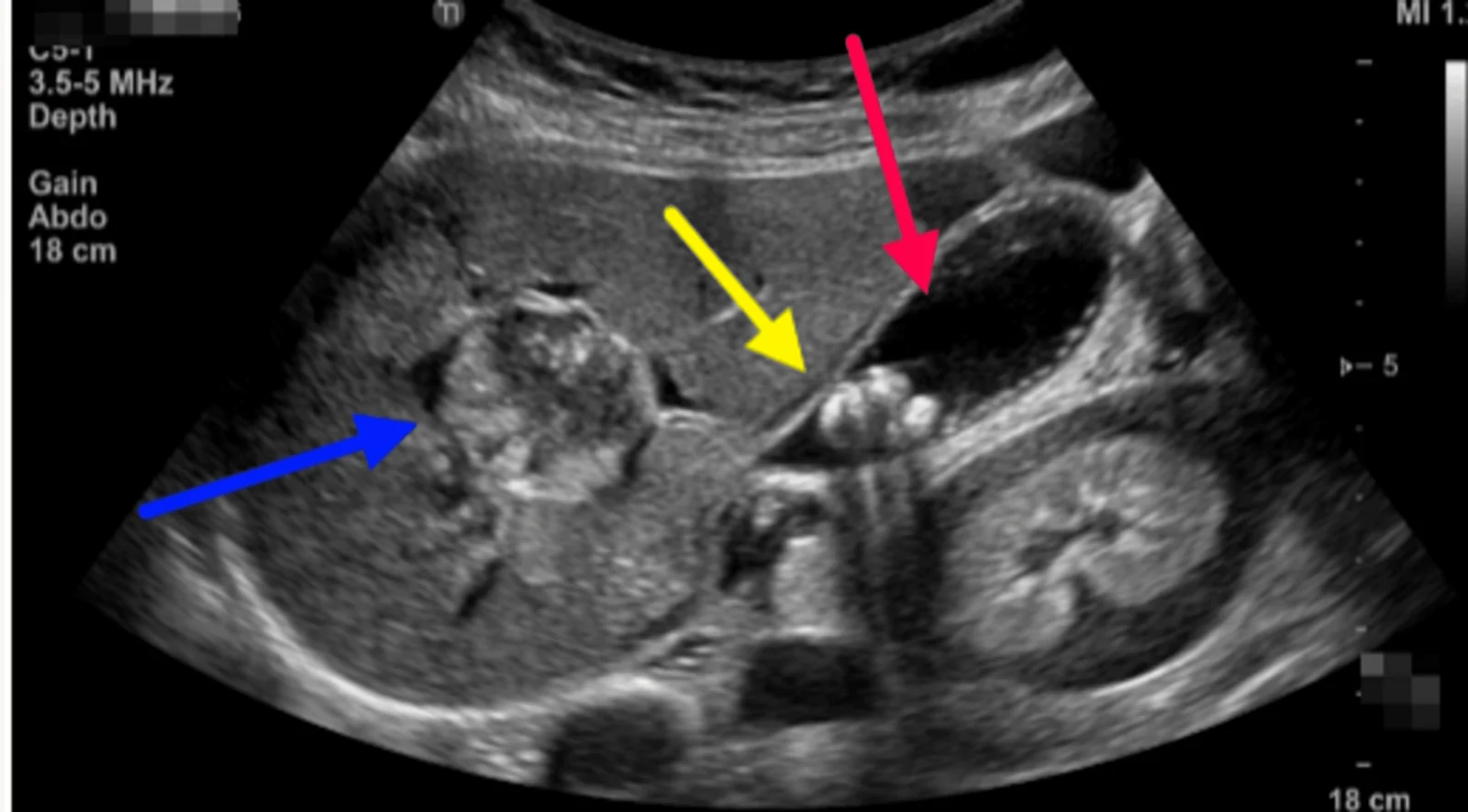
Abdominal Ultrasound (Day 25) Grayscale ultrasonography of the right upper quadrant (longitudinal view) showing a well-defined heterogeneous focal lesion with irregular, lobulated margins (blue arrow) in segment III of the left lobe, measuring 3.4 × 2.3 cm. The gallbladder (GB) (red arrow) contains echogenic calculi (up to 9 mm, yellow arrow) with posterior shadowing and sludge. The right kidney (RK) shows increased cortical echogenicity with reduced corticomedullary differentiation, indicating grade II nephropathy.

In view of the presumed choledocholithiasis, the patient underwent ERCP on Day 37, under light anesthesia. The ampulla was bulging and cannulated without difficulty. However, the procedure revealed a distal CBD stricture with a dilated proximal duct and intrahepatic biliary channels, and no choledocholithiasis was visualized. Critically, no tissue sampling - such as brush cytology, intraductal biopsy, or endoscopic ultrasound-guided fine-needle aspiration - was performed at the time of the stricture identification. A limited sphincterotomy was performed, and a 7 cm, 10 French polyethylene stent was placed across the stricture, resulting in free flow of bile and hemostasis. Fluoroscopic oblique-view ERCP image demonstrating a side-viewing duodenoscope in the second part of the duodenum, with simultaneous dilation of the CBD and main pancreatic duct, consistent with the double-duct sign (Figure 3).

**Figure 3:**
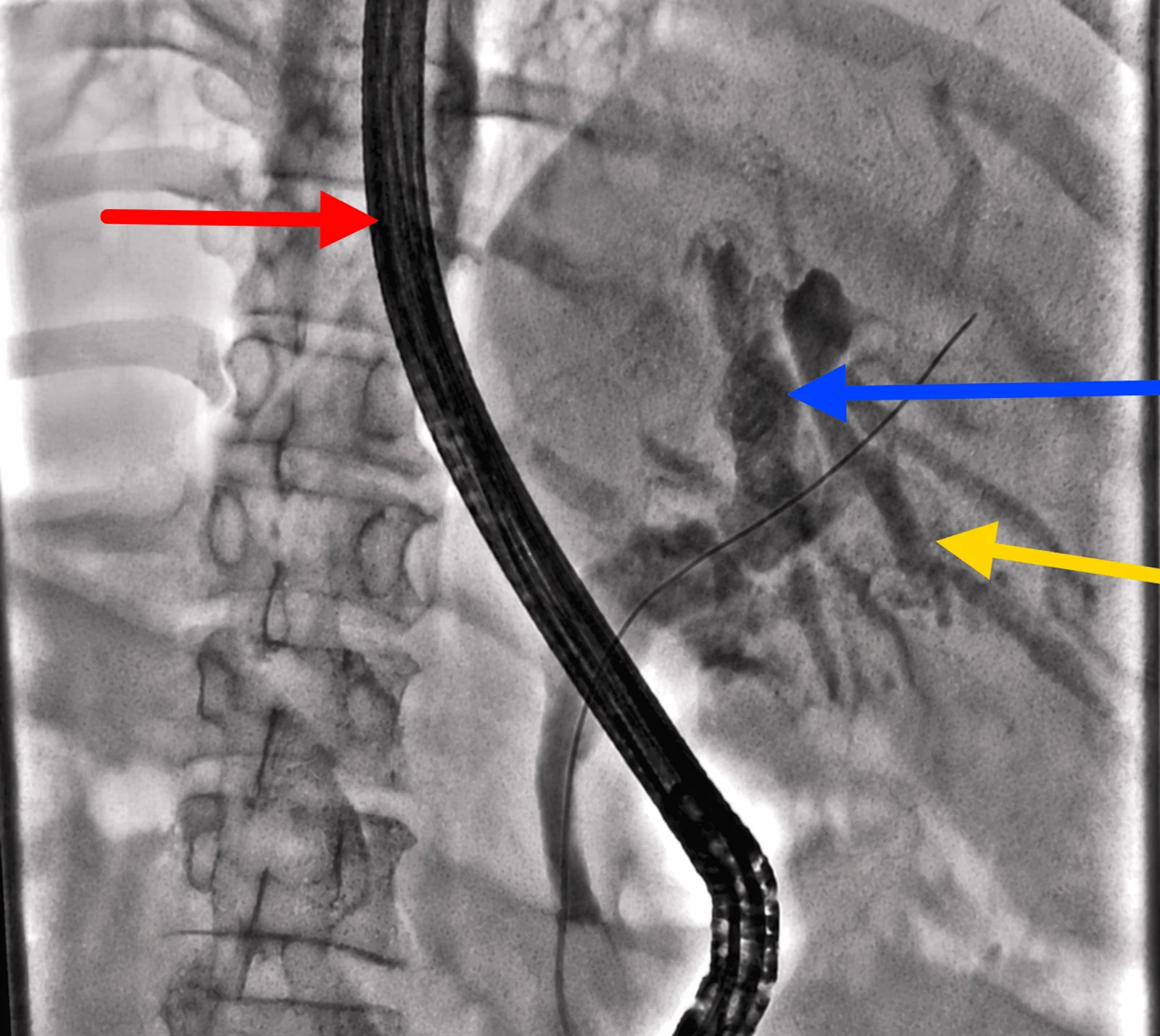
Endoscopic Retrograde Cholangiopancreatography (ERCP) (Day 37) Fluoroscopic image (oblique view) obtained during ERCP, demonstrating a side-viewing duodenoscope (red arrow) in the second part of the duodenum. The cholangiogram reveals a dilated common bile duct (blue arrow) with a smooth but abrupt distal strict with dilated main pancreatic duct (yellow arrow).

The patient reported initial improvement in jaundice and pruritus, but his symptoms recurred within two to three weeks. A preoperative assessment form documented around this period revealed tachycardia (pulse 148/min), hypotension (blood pressure 100 mmHg systolic), and a history of fever and cough for the preceding five days, suggestive of possible cholangitis or systemic inflammatory response, along with evidence of blood sugar monitoring, further supporting the suspicion of new-onset diabetes. However, no HbA1c or glucose values were documented, so diabetes remained suspected rather than confirmed.

The diagnosis remained elusive until tumor marker evaluation was finally obtained on Day 138. Serum CA 19-9 was markedly elevated at greater than 500 U/mL (normal < 37), while alpha-fetoprotein (AFP) was normal at 1.98 IU/mL (normal 0.0-6.05). A normal AFP does not exclude hepatocellular carcinoma (HCC); it only makes HCC less likely. CA 19-9 was interpreted as supportive rather than diagnostic, especially in the setting of biliary obstruction. A repeat triphasic CT scan performed on Days 148-149 confirmed the clinical-radiological diagnosis of stage IV pancreatic malignancy, presumed pancreatic adenocarcinoma: a heterogeneously enhancing mass measuring 43 × 45 mm was identified in the head of the pancreas, causing marked dilatation of both the pancreatic duct and the biliary tree - the classic “double-duct” sign - with associated parenchymal calcifications suggestive of underlying chronic pancreatitis. Multiple hypodense focal lesions were diffusely scattered throughout the liver, consistent with metastatic deposits, and a few small nodules were noted in the visualized lung bases, suspicious for pulmonary metastases. Mild ascites was again present, likely malignant in nature, while the gallbladder remained distended with no radiodense calculi (Figure 4). No histopathological confirmation of the pancreatic or hepatic lesions had been obtained at this point.

**Figure 4:**
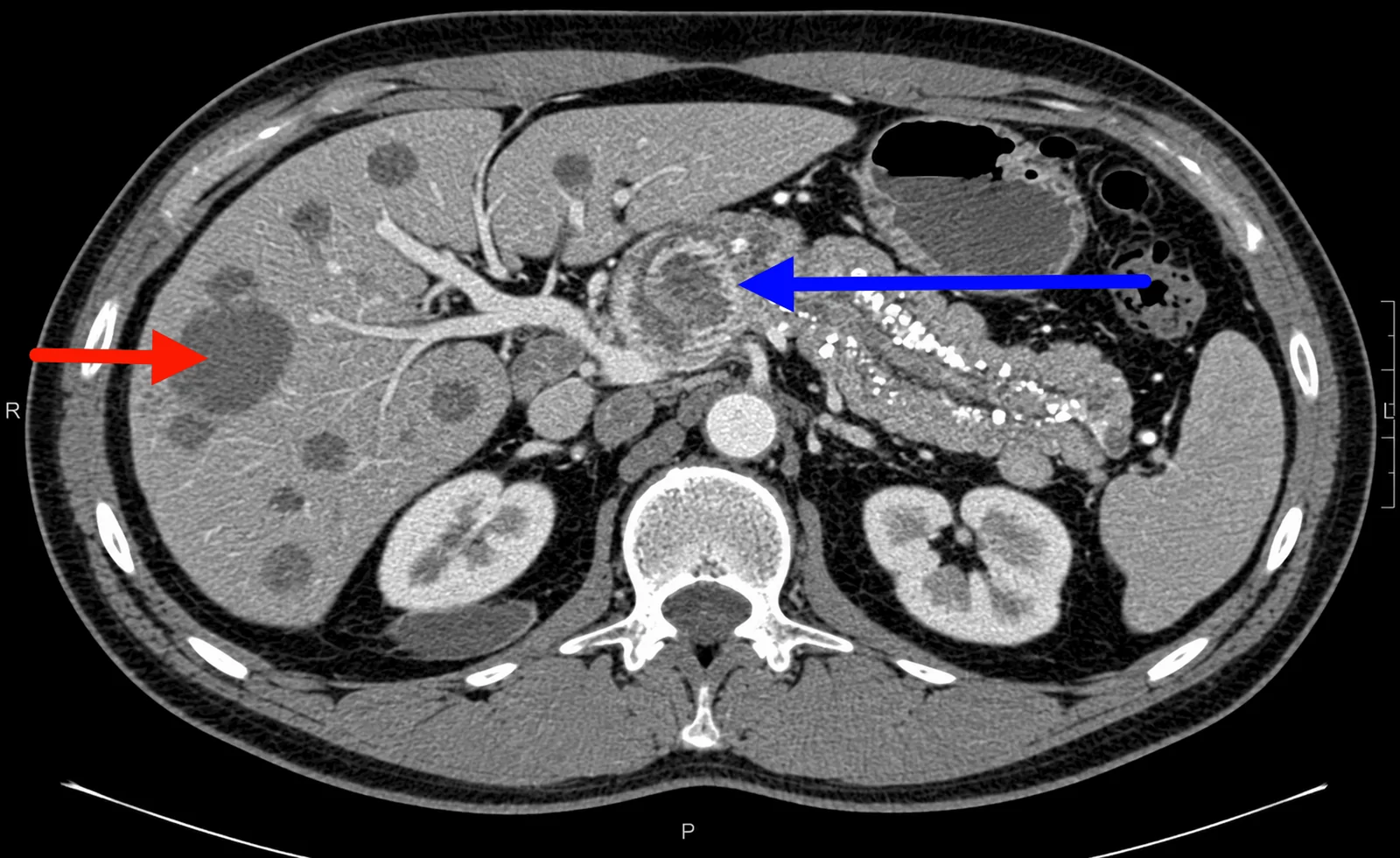
Repeated Triphasic CT Abdomen (Days 148-149) Contrast-enhanced CT image (portal venous phase) confirming clinical-radiological stage IV disease, presumed pancreatic adenocarcinoma. A heterogeneously enhancing mass is seen in the head of the pancreas (blue arrow), measuring 43 × 45 mm, causing marked dilatation of the intrahepatic biliary channels and the main pancreatic duct (classic “double-duct” sign). Multiple hypodense focal lesions are diffusely scattered throughout the liver parenchyma (red arrow), consistent with metastatic deposits. Mild perihepatic ascites is also noted. Pancreatic calcifications are present, suggesting underlying chronic pancreatitis.

Following this clinical-radiological diagnosis of presumed metastatic pancreatic adenocarcinoma, the patient was promptly referred to the oncology service for multidisciplinary management. A comprehensive treatment plan was formulated, taking into account the patient’s young age, performance status, and the extent of metastatic disease. At the time of diagnosis, the patient’s Eastern Cooperative Oncology Group (ECOG) performance status [11] was assessed as 2, given his significant cachexia, persistent jaundice, and mild ascites, but he remained ambulatory and capable of self-care. The multidisciplinary tumor board, comprising medical oncology, gastroenterology, radiation oncology, and palliative care specialists, recommended a palliative systemic chemotherapy approach, given the unresectable nature of the disease with synchronous liver and lung metastases. Considering the patient’s hyperbilirubinemia (total bilirubin 10.4 mg/dL) and deranged liver synthetic function (albumin 2.9 g/dL), the full-dose FOLFIRINOX regimen (oxaliplatin, irinotecan, fluorouracil, and leucovorin) was deemed too hepatotoxic. Full-dose FOLFIRINOX was considered relatively contraindicated because severe hyperbilirubinemia and hypoalbuminemia increase the risk of chemotherapy-related hepatotoxicity, and safety data for full-dose FOLFIRINOX in this setting are limited. A modified regimen of gemcitabine (1000 mg/m^2^) combined with nab-paclitaxel (125 mg/m^2^) was initiated on Day 159, with a dose reduction of 20% for both agents due to baseline cholestasis, along with aggressive supportive care including antiemetics, growth factor support, and nutritional supplementation with pancreatic enzyme replacement. The biliary stent remained patent, and liver function tests were monitored weekly to guide dose adjustments.

The patient’s clinical course during the first two cycles of chemotherapy demonstrated a partial biochemical response, with total bilirubin declining from 10.4 mg/dL to 5.8 mg/dL, alkaline phosphatase falling from 1394 U/L to 620 U/L, and a modest improvement in performance status from ECOG 2 to ECOG 1. He reported a significant reduction in abdominal pain, weight stabilization, and resolution of fever and pruritus. However, a restaging CT scan, as shown in Figure 5, performed after three cycles of therapy (Day 235) revealed a mixed response: the primary pancreatic mass had slightly decreased in size from 43 × 45 mm to 38 × 40 mm, but the liver metastases had increased in number and size, with the largest lesion now measuring 4.2 cm, and the pulmonary nodules had progressed from 5 mm to 12 mm in diameter.

**Figure 5:**
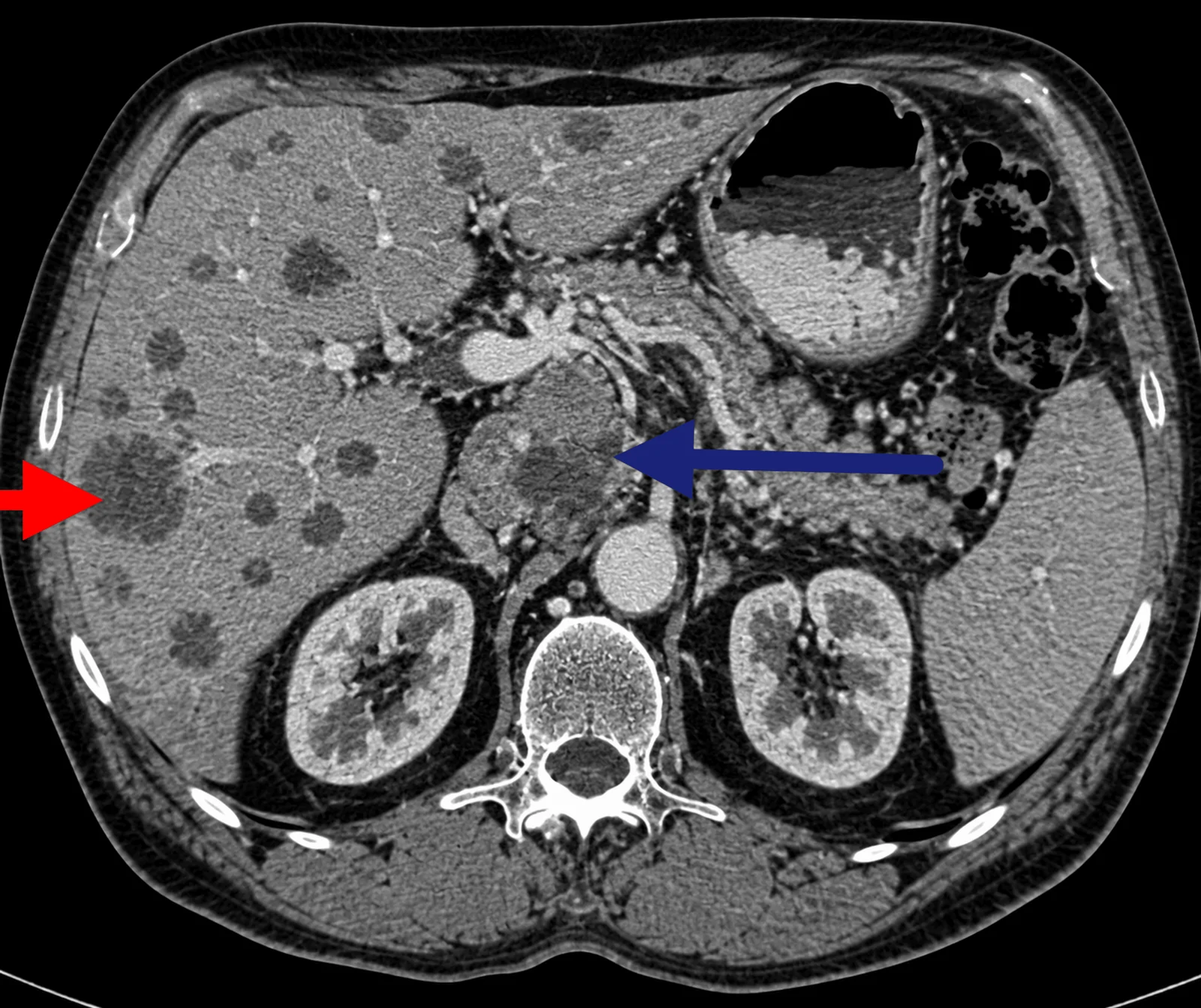
Restaging CT Scan Abdomen Pelvis With IV Contrast (Day 235) The blue arrow indicates the primary pancreatic mass, which slightly decreased from 43 × 45 mm to 38 × 40 mm. The red arrowhead shows metastatic hepatic lesions, which increased in number and size.

Additionally, the malignant ascites had increased, requiring therapeutic paracentesis (approximately 2.5 liters removed on Day 237). Cytological analysis confirmed malignant cells consistent with adenocarcinoma. Cell-block preparation was performed, but immunocytochemistry was not available; therefore, the cytology confirmed metastatic adenocarcinoma but did not independently establish the pancreas as the primary site. Given this disease progression on first-line therapy, the patient was transitioned to second-line chemotherapy with nanoliposomal irinotecan (Onivyde) in combination with 5-fluorouracil and leucovorin, based on the NAPOLI-1 trial regimen [13], starting on Day 256. Concomitantly, palliative radiation therapy to the pancreatic mass (30 Gy in 10 fractions) was administered for pain control, which provided significant symptomatic relief. The patient underwent serial monitoring with tumor markers; CA 19-9 had initially declined to 320 U/mL after the first two cycles but subsequently rose to 780 U/mL by Day 270, confirming disease progression.

By Day 280, the patient’s clinical condition began to deteriorate significantly. He developed worsening cachexia with a weight loss of an additional 10 kg (total weight now 60 kg from a baseline of 76 kg), progressive malignant ascites requiring weekly paracentesis (removing 3-4 liters per session), and persistent fatigue. A PET-CT scan, as shown in Figure 6, performed on Day 322, demonstrated widespread metastatic progression involving new lesions in the peritoneum and bilateral adrenal glands and an increase in the size of the primary mass to 50 × 48 mm, with complete encasement of the superior mesenteric artery and portal vein thrombosis, indicative of locally advanced disease with vascular invasion.

**Figure 6:**
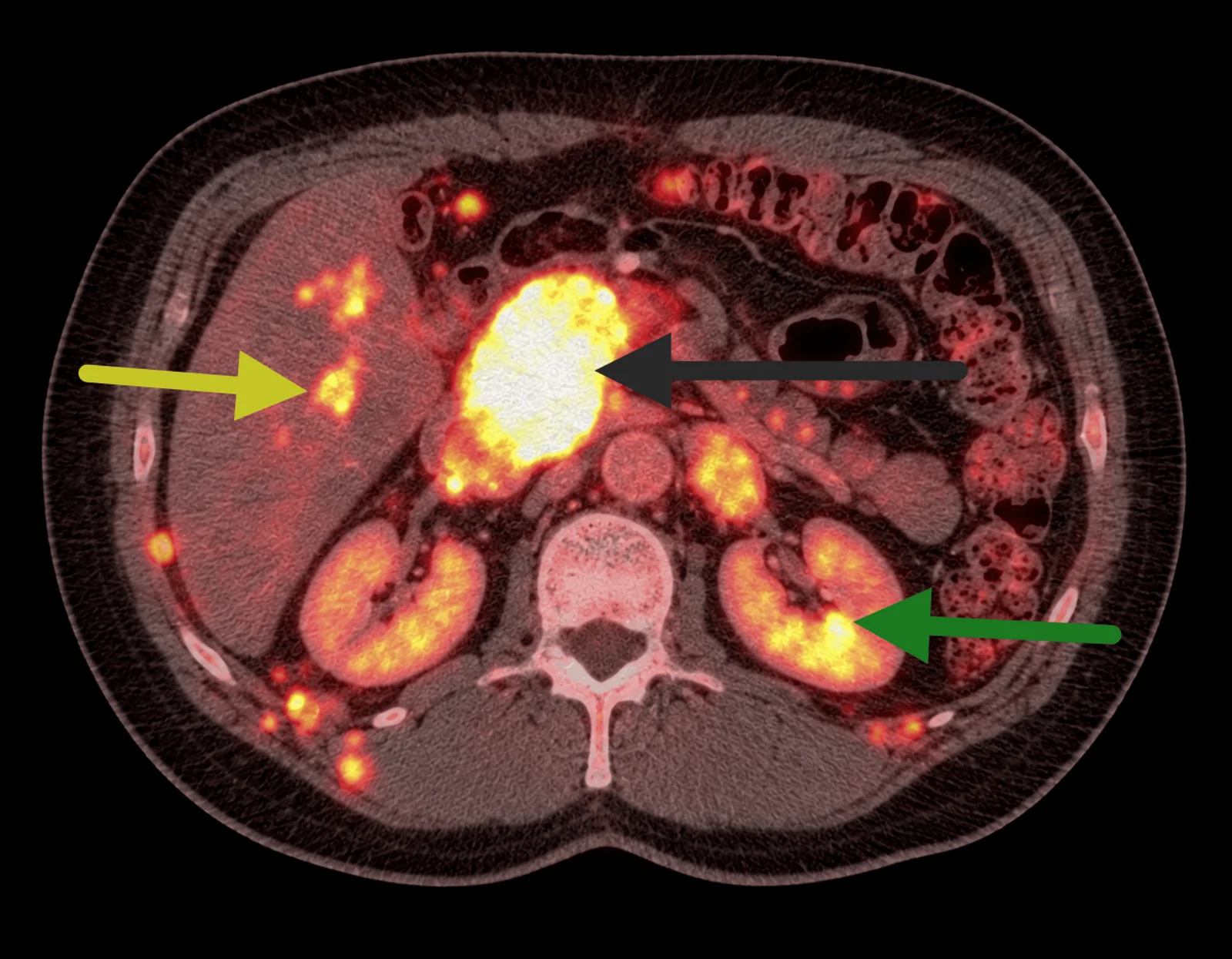
PET-CT Scan (Day 322) - Widespread Metastatic Progression The black arrow showed the primary pancreatic mass: increased to 50 × 48 mm. The yellow and green arrows showed metastatic hepatic and adrenal focal lesions, respectively.

His performance status declined to ECOG 3, and he was no longer a candidate for further systemic chemotherapy. The focus of care transitioned to best supportive care, including optimized pain management with continuous morphine sulphate infusion (titrated to 20 mg/hour), pancreatic enzyme replacement therapy (Creon 25,000 units per meal), nutritional support with enteral feeding via nasogastric tube, and regular paracentesis for ascites management. Additionally, the patient developed progressive renal impairment (creatinine rising to 2.1 mg/dL and urea to 68 mg/dL) consistent with worsening prerenal azotemia and obstructive nephropathy from the portal vein thrombosis and tumor burden, along with persistent grade II nephropathy documented on follow-up ultrasound. The oncology team, in conjunction with nephrology and palliative care, managed his metabolic derangements, electrolyte imbalances, and anemia (hemoglobin dropping to 8.5 g/dL, requiring periodic blood transfusions).

The patient was admitted to the inpatient palliative care unit on Day 365 due to progressive dyspnea, worsening ascites, and significant pain. He developed progressive respiratory failure secondary to pulmonary metastases and pleural effusion (demonstrated on chest X-ray in Figure 7), requiring supplemental oxygen therapy (2-4 liters per minute via nasal cannula).

**Figure 7:**
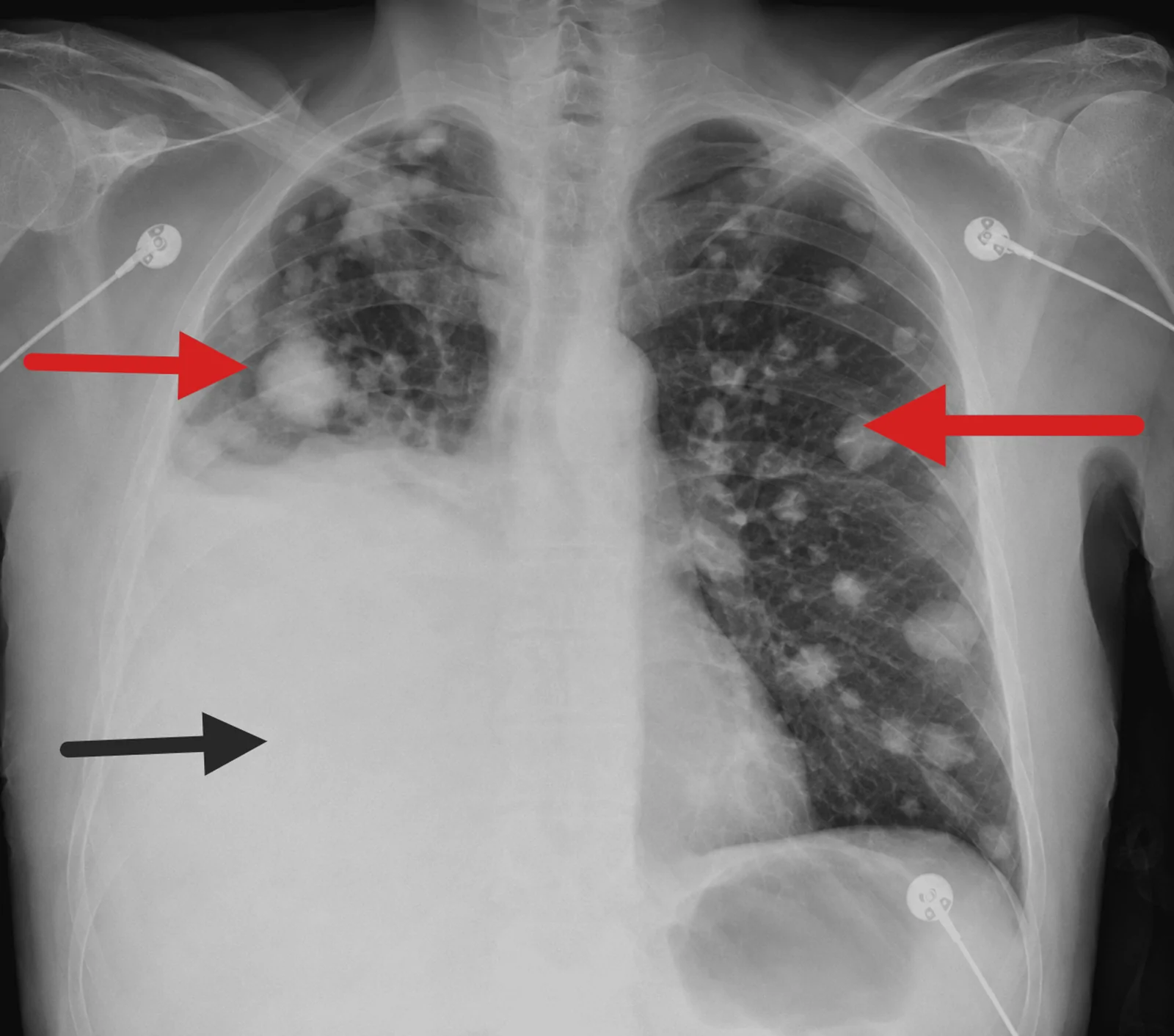
Chest X-Ray - Pulmonary Metastases and Pleural Effusion The black arrow shows right malignant pleural effusion, and the red arrows show multiple pulmonary metastatic lesions.

Despite aggressive supportive measures, his clinical status continued to decline, and he developed hepatic encephalopathy with asterixis, altered sensorium, and a declining Glasgow Coma Scale to 12/15, attributed to progressive hepatic dysfunction and portosystemic shunting from the extensive tumor burden. The patient remained comfortable with the involvement of a dedicated palliative care team, and his family was counseled regarding the poor prognosis. The patient expired on Day 372, approximately 7.5 months after the clinical-radiological diagnosis of presumed stage IV pancreatic adenocarcinoma and 12 months from his initial presentation with obstructive jaundice. The cause of death was multiorgan failure secondary to metastatic pancreatic adenocarcinoma, with contributions from progressive hepatic failure, respiratory compromise, and malignant cachexia. No autopsy was performed per the family’s request.

This case represents a complete clinical trajectory from initial presentation through clinical-radiological diagnosis, active oncological management, disease progression, and end-of-life care, highlighting the aggressive nature of pancreatic adenocarcinoma even in younger patients and the importance of early recognition of malignant biliary strictures. Written informed consent for the publication of this case report and any accompanying images was obtained from the patient’s next of kin, and a copy is available for review by the journal’s editorial board. The patient’s family was provided with comprehensive bereavement support and counseling following his demise, in accordance with institutional protocols.

## Discussion

This case illustrates a prolonged diagnostic odyssey in a young patient with PDAC, initially attributed to choledocholithiasis. That initial interpretation was followed by a five-month delay before the underlying malignancy was recognized, during which the disease progressed to metastatic status. Several points in the diagnostic pathway warrant attention, particularly the interpretation of the initial imaging, the absence of tissue acquisition during ERCP, and the delayed evaluation of clinical features suggestive of pancreatic malignancy. These issues are particularly relevant when evaluating patients with obstructive jaundice and an unexplained biliary stricture.

Biliary strictures remain diagnostically challenging because a substantial proportion are malignant, most commonly due to pancreatic adenocarcinoma or cholangiocarcinoma [10,14]. Distinguishing malignant from benign strictures, including those associated with choledocholithiasis or chronic pancreatitis, can be difficult on imaging alone [15]. In the present case, the initial CT demonstrated a markedly dilated CBD, measuring 16 mm at the porta hepatis, together with multiple dense intraluminal foci in the duodenum that were interpreted as passed gallstones. Although this interpretation was plausible in the clinical context, it appears to have shifted attention away from the persistent biliary obstruction. The patient's young age may also have contributed to a lower initial suspicion of malignancy [3]. This is clinically important because pancreatic cancer in younger adults is frequently associated with diagnostic delays, partly because nonspecific symptoms are attributed to more common benign conditions [3,16].

The subsequent CT performed on Days 148-149 demonstrated the classic double-duct sign, characterized by simultaneous dilatation of the CBD and main pancreatic duct [17]. In the appropriate clinical setting, particularly in a patient with obstructive jaundice, this finding should prompt careful evaluation for an underlying pancreatic or periampullary malignancy [17]. Reported sensitivity and specificity of the double-duct sign vary according to the imaging modality and patient population. Still, its diagnostic significance is substantially greater in patients with jaundice or a pancreatic mass [18]. In this case, recognition of the evolving ductal abnormalities at an earlier stage might have prompted a more definitive evaluation and potentially shortened the diagnostic interval.

The delayed assessment of tumor markers represents another missed opportunity, although CA 19-9 should not be interpreted as a stand-alone diagnostic test [6]. Serum CA 19-9 was not obtained until Day 138, approximately five months after the initial presentation, and was markedly elevated at >500 U/mL, whereas AFP was normal. CA 19-9 has recognized limitations, particularly in the setting of biliary obstruction, where elevations may occur in the absence of malignancy [6,19]. Accordingly, its use as a screening test in asymptomatic individuals is not recommended [7]. Nevertheless, in a symptomatic patient with persistent obstructive jaundice and an unexplained biliary stricture, a markedly elevated CA 19-9 should increase clinical suspicion and support expedited diagnostic evaluation [6]. Importantly, CA 19-9 is better regarded as an adjunct to imaging and tissue diagnosis rather than a substitute for histopathological confirmation [7,20].

Perhaps the most consequential missed opportunity occurred during the ERCP performed on Day 37. The procedure demonstrated a distal CBD stricture without evidence of choledocholithiasis; however, no brush cytology, fluoroscopically guided intraductal biopsy, or EUS-guided tissue acquisition was performed [10,21]. A 7-cm, 10-French polyethylene stent was subsequently placed to relieve the obstruction. Although biliary drainage was appropriate for managing the obstruction, stenting without obtaining tissue left the underlying cause unresolved and contributed to a subsequent delay in oncological assessment [22].

Current guidance emphasizes the importance of tissue acquisition when the etiology of a biliary stricture remains uncertain [10,21]. The American Society for Gastrointestinal Endoscopy (ASGE) recommends combining ERCP-based brush cytology with fluoroscopically guided biopsy for indeterminate biliary strictures rather than relying on brush cytology alone [10]. The 2023 ASGE guideline also recognizes a role for cholangioscopy-guided and EUS-guided sampling, particularly when initial sampling is nondiagnostic [21]. EUS-guided tissue acquisition is particularly relevant for distal biliary strictures because it can simultaneously evaluate the pancreas and adjacent structures [23]. Although brush cytology is highly specific, its sensitivity remains limited, making a multimodal diagnostic approach preferable [24]. In the present case, the absence of tissue acquisition during ERCP represented a significant diagnostic gap and was followed by an approximately three-month delay in oncological management.

The case also highlights the increasing clinical importance of pancreatic cancer in younger adults [3,16]. Although PDAC remains predominantly a disease of older individuals, incidence among younger populations has been increasing, underscoring the need to avoid dismissing malignancy solely on the basis of age [16]. Early manifestations such as abdominal pain, weight loss, and alterations in glucose metabolism are often nonspecific and may be attributed to more common disorders [25]. In this patient, blood sugar monitoring during hospitalization suggests that abnormalities in glucose metabolism were already being observed. New-onset diabetes is a recognized clinical association of pancreatic cancer and may precede its diagnosis by several years [12,25]. In the presence of concurrent weight loss, abdominal symptoms, and unexplained biliary obstruction, such findings should prompt consideration of an underlying pancreatic process, even in a younger patient [26]. In this case, this warning signal does not appear to have been pursued with sufficient diagnostic rigor.

The case should, however, be interpreted in light of several limitations. Histopathological confirmation of the pancreatic and hepatic lesions was not obtained because the patient was lost to follow-up before biopsy could be performed. Consequently, the diagnosis of metastatic disease was based on the clinical and radiological findings available at the time. Genetic testing, including assessment for potentially relevant alterations such as BRCA1/2 and KRAS, was also not performed, limiting assessment of the patient's underlying molecular and hereditary risk profile [27]. The available family history was limited, and formal genetic counseling was not documented. In addition, the observational nature of a single case does not establish a causal relationship between any individual diagnostic decision and the subsequent clinical outcome. The reported survival estimates for advanced PDAC also cannot be directly applied to an individual patient.

Several practical lessons emerge from this case. First, an unexplained distal biliary stricture, particularly in the absence of definite choledocholithiasis, should prompt careful exclusion of malignancy regardless of the patient's age [10,28]. Second, a double-duct sign in a patient with obstructive jaundice warrants timely assessment for pancreatic or periampullary malignancy [17,29]. Third, CA 19-9 can provide useful supportive information in symptomatic patients but should be interpreted cautiously in the setting of biliary obstruction and should never replace tissue diagnosis [6,30]. Fourth, new-onset diabetes accompanied by weight loss, abdominal pain, or other concerning features should increase suspicion for pancreatic malignancy [12,25]. Finally, young age should not substantially lower the threshold for investigating persistent or unexplained biliary obstruction when clinical and radiological findings remain concerning [3,16].

## Conclusions

This case demonstrates how pancreatic adenocarcinoma may initially mimic choledocholithiasis, particularly in a younger patient in whom the pretest suspicion for malignancy may be lower. The combination of persistent biliary obstruction, a distal CBD stricture, progressive ductal dilatation, a double-duct sign, and markedly elevated CA 19-9 should prompt urgent evaluation for pancreatic malignancy. The diagnosis in this case was a clinical-radiological diagnosis of presumed metastatic pancreatic adenocarcinoma, supported by ascitic cytology showing malignant adenocarcinoma but without immunocytochemical confirmation of pancreatic origin. Early tissue acquisition during ERCP or through EUS-based approaches may have reduced the diagnostic delay in this case. CA 19-9 should be regarded as an adjunct rather than a diagnostic or screening test, particularly in the setting of biliary obstruction. More broadly, adherence to established recommendations for the evaluation of indeterminate biliary strictures and maintaining an appropriate level of suspicion for pancreatic cancer irrespective of age may help prevent similar delays in diagnosis. Earlier tissue acquisition may have reduced diagnostic delay, but this single case cannot establish that it would have changed survival or outcome.
